# Supplementary material for: Struggles and strategies in anaerobic and aerobic cycling tests: A mixed-method approach with a focus on tailored self-regulation strategies
Source: PLoS One. 2021 Oct 27;16(10):e0259088. doi: 10.1371/journal.pone.0259088 (PMC8550367; doi:10.1371/journal.pone.0259088)
Supplement: S1 Table — A. Obstacles the participants reported to have experienced during the anaerobic / aerobic test. Note. * refers to obstacles that were only mentioned after the anaerobic test, while ** references obstacles that were only mentioned after the aerobic test. B. Strategies used (thoughts, sensations or behaviors) during the anaerobic / aerobic test. Note. * refers to strategies that were only mentioned after the anaerobic test, while ** references strategies that were only mentioned after the aerobic test. C. Potential strategies reported by participants after the anaerobic / aerobic test. Note. * refers to potential strategies that were only mentioned after the anaerobic test, while ** references potential strategies that were only mentioned after the aerobic test. (DOCX) [file pone.0259088.s001.docx]

**S1A Table. Obstacles the participants reported to have experienced during the anaerobic / aerobic test.**

| **General Theme** | **Category** | **Description** | **Example** |
| --- | --- | --- | --- |
| **Missing focus** | Distraction by screen* | Looking at the screen too often or getting distracted by the display. | *“slow down 🡪 accelerate again; towards the end (effort) look at display, time display”* |
|  | Distraction through thoughts* | Being distracted, other thoughts ensure that the focus on the task is not given. | *“distracting thoughts”* |
|  | Distraction** | Distraction (e.g., Borg) during the task was hindering or there was an inability to distract oneself any longer. | *“looked at sheet, tried to estimate Borg”* |
| **Missing drive** | Incentive | A good performance has no meaning, completing the task is not important enough for the participant. | *“I don’t need this exercise, I am exercising in other ways”* |
|  | Demotivation | Having no intention to wear oneself out, no motivation for the task (aerobic test: or to ride any longer). | *“I don’t feel like it anymore”* |
|  | Duration* | Duration of the test (30 sec.) is perceived as long or too long, which is demotivating. | *“30 seconds last still very long”* |
|  | Thoughts about stopping** | Possibility to stop at any time is perceived as demotivating. | *“motivation was missing; stop when you want to”* |
|  | Performance reduction** | Conscious experience of declining performance is perceived as demotivating. | *“awareness of slowing down, demotivating, no longer stalling”* |
|  | Periods of time** | Certain periods of the test are experienced as demotivating. | *“always second half of the interval”* |
|  | Screen** | Discouraging or demotivating information on the display (e.g., steps). | *“display: I do not reach to the end anyway”* |
| **Negative sensations** | Frustration | Feelings of frustration or anger about performance, about effort, about oneself. | *“frustration because of fast exertion”* |
|  | Shame* | Experiencing shame during physical performance. | *“embarrassing to be out of breath”* |
|  | Failure* | Feelings of failure in performance, not succeeding. | *“legs feel weak but still not at maximum power”* |
|  | Arousal at Start / Finish* | Specific time periods of the test put the participant under pressure. | *“last 10 seconds”* |
|  | Exhaustion* | Not keeping up performance, feeling exhausted. | *“to drop performance in the last 5 seconds, as it got more and more difficult”* |
|  | Exertion | Perception of the task as strenuous; thoughts, sensations, physical states indicate that the task is (becoming) strenuous. | *“hard to pedal, to drag myself to the finish line, I wanted to quit the test”* |
|  | Boredom** | Task is perceived as boring and monotonous. | *“three minutes are long, boring”* |
|  | Pain** | Experiencing pain in various limbs, which is obstructive. | *“thighs hurt”* |
|  | Pressure to perform** | Feeling under pressure, the inability to go on much longer leads to comparing oneself with others and to more pressure. | *“Now comes the next level, hopefully, I can keep up cadence, fear of the next level”* |
| **Test demands** | Surprised by test demands* | Surprised about the resistance during the test. | *“stop pedaling when resistance started”* |
| **Cycling strategy** | Acceleration* | Participant perceives acceleration during the test as not optimal (e.g., should have been stronger). | *“accelerate faster”* |
|  | Power management* | Force distribution is perceived as not optimal, maximum force was not utilized to the fullest. | *“still some power left”* |
|  | Slowing down* | Unable to maintain cadence. | *“looked at the screen at felt maximum power and saw how it dropped”* |
|  | Riding behavior / technique** | Riding technique hindering during the test (e.g., started too fast at the beginning). | *“pedaling too fast”* |
| **Discomfort** | Ergometer* | The settings or conditions of the ergometer are experienced as obstructive. | *“uncomfortable”* |
|  | Posture | Being occupied with (wrong) posture during the test, the position on the bike is arduous.. | *“pain in the saddle; position on the bike”* |
|  | Body | Having difficulties with physical reactions, such as sweating, circulation, which is perceived as uncomfortable. | *“getting nauseous”* |
| **Goal** | Goal achievement** | Having reached the goal or not to having reached the goal. | *“open ending is frustrating; nothing to look forward to as it gets harder and harder”* |
|  | Aimlessness** | There is no fixed, clear goal (open end). | *“no definitive ending”* |
| *Note.* * refers to obstacles that were only mentioned after the anaerobic test, while ** references obstacles that were only mentioned after the aerobic test. | | | |

**S1B Table. Strategies used (thoughts, sensations or behaviors) during the anaerobic / aerobic test.**

| **General Theme** | **Category** | **Description** | **Example** |
| --- | --- | --- | --- |
| **Distancing** | Cut out* | Cutting out everything during the task. | *“to not look on the screen, head down, speed up”* |
|  | Distraction** | Distracting yourself during the task. | *“looking out of the window”* |
| **Attentional focus** | Screen | Concentrating on screen during the task. | *“looking at the screen”* |
|  | Screen: Cadence* | Concentrating on cadence during the task. | *“look at cadence, trying to hold cadence”* |
|  | Screen: Time* | Concentrating on time during the task. | *“to see how the seconds go down”* |
|  | Body* | Focusing on body or specific movements during the task, such as pedaling. | *“gripping the handlebar tight”* |
|  | Focus on test / goal* | Focusing on the task. | *“just think about the test”* |
|  | Concentration** | Focusing oneself. | *“concentrate to achieve steady breathing”* |
|  | Technique** | Focusing on riding technique (e.g., cadence). | *“pulling instead of pedaling”* |
| **Drive** | Exertion | Motivating to feel the effort, effort is perceived as something positive. | *“exertion is progress”* |
|  | Ambition | Being ambitious, showing it, pushing oneself, motivated by ambition. | *“deliver best performance”* |
|  | Imagination | Thinking of a similar / relaxing context (e.g., a bicycle race). | *“imagine riding up a mountain, when it got hard”* |
|  | Motivation | Having motivation for the task. | *“motivated to exercise”* |
|  | Rationalization* | Keeping in mind what the task is useful for (e.g., to exercise more again). | *“exercise is good, healthy for the body”* |
|  | Joy | Enjoying the task or the effort. | *“joy in exertion”* |
|  | Self-Encouragement | Cheering oneself on. | *“you can do it. It’s only 30 seconds”* |
|  | Pride | Being satisfied with yourself and your own performance. | *“to be proud of oneself when exercising”* |
|  | Self-Worth** | Approaching the task with confidence. | *“comparison with situations, where I performed successfully, more than possible”* |
|  | Flow** | Having your own rhythm (flow). | *“stepped slower at the beginning, slowly increased until finding rhythm”* |
|  | Attitude** | Positive attitude towards the task. | *“exercise makes you happy”* |
| **Comfort** | Position** | Changing posture / position. | *“change arm position after every level”* |
| **Miscellaneous** | Miscellaneous** | Items in this category are not assignable to the other categories. | *“fear as incentive, shame”* |
| **Planning** | Planning** | Planning the course of the test. | *“just finishing a couple of legs”* |
| **Pressure to performance** | Easiness* | Do not put pressure on yourself during the task. | *“it’s ok to cycle like that”* |
|  | Sense of duty* | Feeling a commitment to the study. | *“commitment to study”* |
| **Goal** | Goal setting** | Setting a precise goal. | *“reaching the next level as goal”* |
|  | Goal focus** | Focusing on the goal. | *“always finish intervals, focusing on the respective interval”* |
| *Note.* * refers to strategies that were only mentioned after the anaerobic test, while ** references strategies that were only mentioned after the aerobic test. | | | |

**S1C Table. Potential strategies reported by participants after the anaerobic / aerobic test.**

| **General Theme** | **Category** | **Description** | **Example** |
| --- | --- | --- | --- |
| **Distancing** | Distraction | Consciously thinking about something else. | *“focus on different body part, looking out at the lake”* |
|  | Cut out thoughts | Cutting out all thoughts. | *“not thinking about being uncomfortable”* |
|  | Imagination | Imagining something alternative, such as a different environment* or something distracting, like motivating music**. | *“imagine being outside”* |
| **Attentional Focus** | Screen | Focusing on the screen, on the display. | *“looking at screen -> wanting to proceed on display”* |
|  | Body | Focusing on body and specific movements / extremities. | *“looking at thighs and feet, concentrate on legs more”* |
|  | Screen: Cadence** | Focusing on cadence. | *“cycling constantly, holding cadence, focus”* |
|  | Screen: Time** | Focusing on time. | *“focusing on the countdown”* |
|  | Technique** | Focusing on a specific riding technique. | *“focus on pushing and pulling the pedals”* |
|  | Concentration** | Concentrating (on the task). | *“being focused”* |
| **Drive** | Ambition | Ambition as a motivator to be as good as possible. | *“outperform yourself”* |
|  | Attitude | Approaching the task with a positive attitude (e.g., task as a challenge). | *“perceiving exertion as positive”* |
|  | Imagination | Imagining motivating cycling scenes, such as a finish line or motivating things. | *“imagining cycling with buddy, catching up with him”* |
|  | Motivation through nice thoughts* | Motivating thoughts of something nice (e.g., an upcoming vacation). | *“thinking about the nice weekend”* |
|  | Rationalization | Playing down / Talking up the task (e.g., exercising is healthy). | *“sweating is healthy”* |
|  | Self-Encouragement | Cheering yourself on to make it, for getting it done. | *“I can do more than I give myself credit for”* |
|  | Screen** | Motivating oneself effectively with the display on the screen. | *“Watch time bar km display: motivating, conversion how much already driven”* |
|  | Pride** | Realizing how proud one is of oneself. | *“thinking about the success”* |
| **Performance** | Technique* | Achieving a good performance via technical refinements or a certain attitude. | *“pulling the clip pedals, gripping the handlebar”* |
|  | Orientation on screen: Cadence* | Optimizing performance via display information such as achieved cadence. | *“cadence mustn’t sink, I try to keep on pedaling”* |
|  | Orientation on screen: time* | Optimizing performance via display information such as time elapsed. | *“looking at the timeline, half is already done”* |
|  | Adjustment** | Optimizing performance via display information or additional acceleration. | *“only to 75, do not cycle faster, rather keep going longer”* |
|  | Take off pressure** | Taking pressure off yourself, being satisfied with oneself. | *“cadence is alright, just as good as a faster one”* |
|  | Pressure to perform** | Participant does not want to fail. | *“Not wanting to fail, not wanting to be a drop-out; settling in, slow start”* |
| **Planning** | Planning* | Mentally planning the course of the test and preparing for difficult moments. | *“control breathing, do not breathe hectically when resistance hits”* |
| **Goal** | Goal achievement* | Wanting to achieve a certain goal. | *“concentrate on the goal”* |
|  | Goal setting | Setting a specific goal or intermediate goals. | *“cycling at least 15 minutes, reaching a cadence of 75 one last time”* |
|  | Goal focus** | Focusing on a goal or intermediate goals. | *“focus on making it”* |
| *Note.* * refers to potential strategies that were only mentioned after the anaerobic test, while ** references potential strategies that were only mentioned after the aerobic test. | | | |
