# Supplementary material for: Struggles and strategies in anaerobic and aerobic cycling tests: A mixed-method approach with a focus on tailored self-regulation strategies
Source: PLoS One. 2021 Oct 27;16(10):e0259088. doi: 10.1371/journal.pone.0259088 (PMC8550367; doi:10.1371/journal.pone.0259088)
Supplement: S2 Table — (DOCX) [file pone.0259088.s002.docx]

**S2 Table. If- and then-components in categories (incl. frequency) used by participants to enhance performance in the anaerobic / aerobic test.**

| **Test** | **Category of if-component** | **Freq.** | **Category of then-component** | **Freq.** |
| --- | --- | --- | --- | --- |
| **Anaerobic** | Exertion | 22 | Self-Encouragement | 19 |
|  | Start / Finish | 15 | Ambition | 12 |
|  | Distraction through screen | 6 | Planning | 5 |
|  | Frustration | 6 | Distraction | 4 |
|  | Surprised by test demands | 5 | Concentration on screen: Time | 4 |
|  | Acceleration | 3 | Imagination | 4 |
|  | Duration | 3 | Body | 4 |
|  | Demotivation | 3 | Orientation on screen: Cadence | 4 |
|  | Exhaustion | 3 | Concentration on screen | 3 |
|  | Slowing down | 3 | Motivation | 3 |
|  | Body | 2 | Rationalization | 3 |
|  | Power management | 2 | Focus on test / goal | 2 |
|  | Pain | 2 | Technique / Posture | 2 |
|  | Distraction through thoughts | 1 | Concentration on screen: Cadence | 1 |
|  | Incentive | 1 | Attitude | 1 |
|  | Posture | 1 | Cutting out thoughts | 1 |
|  |  |  | Motivation through nice thoughts | 1 |
|  |  |  | Orientation on screen: Time | 1 |
|  |  |  | Pride | 1 |
|  |  |  | Goal achievement | 1 |
|  |  |  | Goal focus | 1 |
|  |  |  | Goal setting | 1 |
| **Aerobic** | Exertion | 24 | Goal setting | 18 |
|  | Periods of time | 14 | Distraction | 16 |
|  | Demotivation | 9 | Technique | 11 |
|  | Boredom | 6 | Self-Encouragement | 9 |
|  | Screen | 5 | Ambition | 7 |
|  | Pain | 5 | Imagination | 4 |
|  | Frustration | 4 | Body | 4 |
|  | Performance reduction | 4 | Goal focus | 4 |
|  | Body | 3 | Screen | 2 |
|  | Not defined | 3 | Concentration | 2 |
|  | Distraction | 2 | Posture | 2 |
|  | Thoughts about stopping | 2 | Rationalisation | 2 |
|  | Pressure to perform | 2 | Concentration on screen: Cadence | 1 |
|  | Riding behavior / technique | 2 | Concentration on screen: Time | 1 |
|  | Goal achievement | 2 | Take off pressure | 1 |
|  | Posture | 1 | Attitude | 1 |
|  | Incentive | 1 | Joy | 1 |
|  |  |  | Motivation | 1 |
|  |  |  | Miscellaneous | 1 |
|  |  |  | Pride | 1 |
