# Supplementary material for: Struggles and strategies in anaerobic and aerobic cycling tests: A mixed-method approach with a focus on tailored self-regulation strategies
Source: PLoS One. 2021 Oct 27;16(10):e0259088. doi: 10.1371/journal.pone.0259088 (PMC8550367; doi:10.1371/journal.pone.0259088)

**S1 Fig. Flowchart visualizing the protocol of session one (a) and session two (b). Manipulation means the random assignment to either the goal intention / implementation intention condition.**


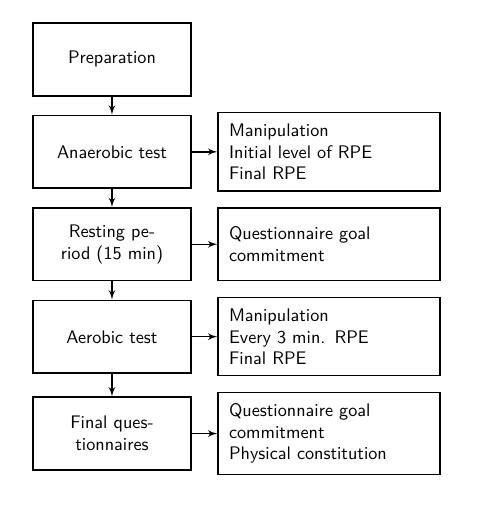


b

a


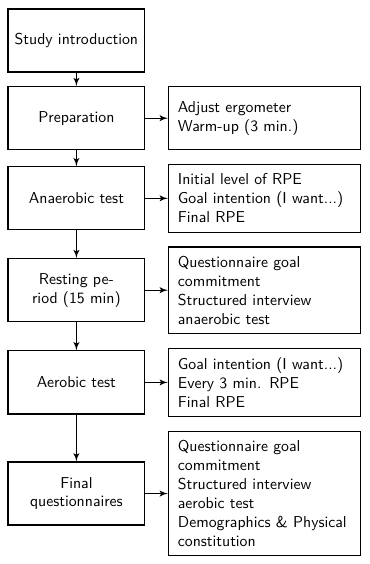

Supplement: S1 Fig — Manipulation means the random assignment to either the goal intention / implementation intention. condition. (DOCX) [file pone.0259088.s003.docx]
