# Supplementary material for: Struggles and strategies in anaerobic and aerobic cycling tests: A mixed-method approach with a focus on tailored self-regulation strategies
Source: PLoS One. 2021 Oct 27;16(10):e0259088. doi: 10.1371/journal.pone.0259088 (PMC8550367; doi:10.1371/journal.pone.0259088)
Supplement: S1 Appendix — (DOCX) [file pone.0259088.s004.docx]

**S1 Appendix. Study Materials**

***A.1 Goal Intention: Preparation for the Test***

Your goal for the next task is **to reach maximum power as fast as possible and hold as long as possible** (aerobic test: **to cycle as long as possible**). Write down your goal:

I want _______________________________________________________!

***A.2 Structured Interview Anaerobic and Aerobic Test***

At the beginning of the task, you set yourself the goal to reach maximum power as fast as possible and hold as long as possible (aerobic test: to cycle as long as possible). However, we never reach our goals to 100% and there is always something that prevents us from doing so. I am interested in what helped you during the task to achieve your goals as good as possible, what has prevented you from reaching your goals or what you could have done differently in order to achieve your goals even better. This concerns your thoughts, sensations, or your behavior. There is no right and wrong, perhaps there are some things you may come to think of more than others. Just answer the questions as good as possible.

Can you spontaneously think of a situation in which a thought, a feeling or a behavior was hindering or would have been useful for reaching the goal? What would be an effective action or thought that would help you to better achieve your goal?

| **Critical situations**  When did the situation arise?  How can the situation be recognized? | **Goal-oriented actions**  How could you act effectively?  What would be an effective thought? |
| --- | --- |
|  |  |
|  |  |

***Guiding Questions***

[ These are asked after the spontaneous query. Examples are given if the subjects do not understand what we mean by thoughts/feelings/behaviours or if the subjects do not know how they could act effectively. First, the subjects should always generate their own ideas.]

Thoughts:

1. Did you have a thought during the task that was helpful for you? When did the thought occur? (*e.g., encouragement*)
2. Were there any situations where an obstructive thought went through your mind? (*e.g., demotivating thoughts, putting yourself under pressure*)
3. What would be an effective action or thought in the situation? (*e.g., motivate yourself, distract yourself, encourage yourself*)

Sensations:

1. Did you have a feeling during the task that helped you? When? (*e.g., ambition*)
2. Were there any situations where you had obstructive sensations? Why did you terminate the exercise? (*e.g., effort, pain, frustration) (normal pain or pulse*)
3. What would be an effective action or thought in the situation? (*e.g., motivate oneself, distract oneself, encourage oneself, target Borg value*)

Behavior:

Did you behave in a certain way that helped you in a situation? When? (*e.g., cheering, distracting oneself*)

Were there any situations where your behavior hindered you?... (*e.g., maximum force too late, too much force left*)

What would be an effective action or thought in the situation? (*e.g., cheering yourself on, never letting up*)

***A.3 Intervention***

***Preparation for the Task***

Analogous to the last session, the goal of the task is **to reach maximum power as fast as possible and hold as long as possible** (aerobic test: **to cycle as long as possible**). Write down your goal:

I want _______________________________________________________!

Additionally, you considered in which situations a thought, feeling or behavior was hindering or would have been useful for reaching the goal in the last session. Also, you have thought about what could be an effective action or effective thought that would help you to better achieve you goal in in the situation. The points you mentioned are listed below:

| **Situations** | **Behaviors/Thoughts** |
| --- | --- |
|  |  |
|  |  |
|  |  |

Please reflect on these situations and behaviors / thoughts for a moment.

**Only implementation intention condition:**

In the next step, you link the situations with appropriate actions or thoughts in very concrete **if** (situation) - **then** (action/thought) **plans**. Write down your plans:

**If** ,

(Situation)

**then** !

(Behavior/Thought)

After you have written down your plans, go through them again at your leisure. If you want, you can also recite them in your mind. Your should have the feeling that you have fully internalized them before you begin the task.
