# Supplementary material for: Struggles and strategies in anaerobic and aerobic cycling tests: A mixed-method approach with a focus on tailored self-regulation strategies
Source: PLoS One. 2021 Oct 27;16(10):e0259088. doi: 10.1371/journal.pone.0259088 (PMC8550367; doi:10.1371/journal.pone.0259088)
Supplement: Attachment — Submitted filename: Response to Reviewers.docx [file pone.0259088.s005.docx]

Universität Konstanz · Fach · 78457 Konstanz

Anna Hirsch

Wissenschaftliche Mitarbeiterin

Department Sportwissenschaft, Sportpsychologie

Universitätsstraße 10

78464 Konstanz

Anna.Hirsch@uni-konstanz.de

www.uni-konstanz.de

Universitätsstraße 10

78464 Konstanz

Deutschland

29.07.2021

Revision for: PONE-D-21-13120

*Dear Mr. Gruet,*

*Thank you for the constructive review concerning our paper entitled “Struggles and Strategies in Anaerobic and Aerobic Cycling Tests: A Mixed-method Approach with a Focus on Tailored Self-Regulation Strategies" submitted to PLOS ONE (Manuscript ID: PONE-D-21-13120).*

*We addressed the remarks raised by the reviewers and changed the manuscript accordingly (for detailed replies to each issue that was raised, please see table below). The revised version of the manuscript is 36 pages long and includes 2 figures and 4 tables (additionally, 5 Tables and 1 Figure in the Supporting information).*

*We again thank you for your helpful review. By incorporating your suggestions, we are confident that the changes we have made to the original version of the paper have improved its quality substantially.* *We have paid particular attention to streamlining and restructuring the introduction so that it is more straightforward in its argumentation of the aims of the study. As you can also see in detail in the response letter, we have resolved the unclarity regarding the RPE measurements and feel that the manuscript has gained in comprehensibility (e.g., regarding its measures).*

*We hope for further positive comments from you, and we would be honored if you were to consider our paper for publication in PLOS ONE.*

*Sincerely,*

*Anna Hirsch, Maik Bieleke, Raphael Bertschinger, Julia Schüler & Wanja Wolff*

| **General comment** | **Our response** |
| --- | --- |
| **Issue 1**   Please ensure that your manuscript meets PLOS ONE's style requirements, including those for file naming. The PLOS ONE style templates can be found at (…) | Thank you for pointing out this important style issue. We made sure to adhere closely to the journal’s requirements. |

**Our response to the comments of reviewer 1:**

| **Reviewers’ comments** | **Our response** |
| --- | --- |
| **Issue 1**   Abstract:  Suggest to rephrase ‘fine-grained investigations’. I think I understand the essence of it, but would suggest to adjust word choice as for some of the demands there are in depth investigations (i.e. mental fatigue), although I agree that the psychology of endurance performance has received relatively little research attention compared to some other sporting domains. Overall, the abstract can be written more clearly to capture the aim of the study better, as well as what was done (i.e. whether the interviews informed the if-then plan of the second visit). | Thank you for your correction. We have edited the abstract and it now reads as follows (p. 2):  *Endurance sports pose a plethora of mental demands that exercisers have to deal with. Unfortunately, investigations of exercise-specific demands and strategies to deal with them are insufficiently researched, leading to a gap in knowledge about athletic requirements and strategies used to deal with them. Here, we investigated which obstacles exercisers experience during an anaerobic (Wingate test) and an aerobic cycling test (incremental exercise test), as well as the strategies they considered helpful for dealing with these obstacles (qualitative analysis). In addition, we examined whether thinking of these obstacles and strategies in terms of if-then plans (or implementation intentions; i.e., “If I encounter obstacle O, then I will apply strategy S!”) improves performance over merely setting performance goals (i.e., goal intentions; quantitative analysis). N = 59 participants (age: M = 23.9 years, SD = 6.5) performed both tests twice in a 2-within (Experimental session: 1 vs. 2) × 2-between (Condition: goal vs. implementation intention) design. Exercisers’ obstacles and strategies were assessed using structured interviews in Session 1 and subjected to thematic analysis. In both tests, feelings of exertion were the most frequently stated obstacle. Motivation to do well, self-encouragement, and focus on the body and on cycling were frequently stated strategies in both tests. There were also test-specific obstacles, such as boredom reported in the aerobic test. For session 2, the obstacles and strategies elicited in Session 1 were used to specify if-then plans. Bayesian mixed-factor ANOVA suggests, however, that if-then plans did not help exercisers to improve their performance. These findings shed novel light into the mental processes accompanying endurance exercise and the limits they pose on performance.* |
| **Issue 2**  Introduction:  1. On the first pages the authors present an overview of the exercise-related psychological obstacles that might affect performance. Although some relevant studies are reviewed I am not entirely clear on what the author’s interpretation is of what comprises obstacles. That is, some of the obstacles that are presented reflect strategies (active distraction, coping with pain). To help improve this section I would encourage the authors to be clearer on what is meant with obstacles and strategies (Birrer & Morgan, 2010 might be a useful resource here) and to provide a more coherent overview of the literature. For example, a study that is missing when discussing strategies in cycling is Blanchfield, Hardy, De Morree, Stainao, & Marcora’s (2014) study on self-talk. | Thank you for your helpful suggestions. We agree that some important papers were missing from our literature review. To address this issue, we incorporated the suggested publications by Birrer and Morgan (2010; Reference #5) and by Blanchfield et al. (2014; Reference #15). Moreover, the introduction now embeds our work in several other relevant studies (new References are #6, #7, #9, #10, #12, #13). Finally, we revised the section to improve understanding and clarity (p. 3-4).  *The specific psychological obstacles that exercisers face during an endurance performance and the strategies they apply to deal with them requires more research as this topic is of great scientific and practical importance (1,2). To understand the limits of endurance performance (i.e., factors that hamper or disrupt endurance performance) and design effective sport psychological interventions, knowledge about the various exercise-related psychological obstacles that might affect performance is crucial (3). Accordingly, a growing body of research focuses on the obstacles that endurance athletes have to deal with during performance (e.g., 1,4) and comprehensive reviews shed light on the characteristics of psychological interventions in sport that allow them to improve endurance performance (e.g., 5).*  *According to the psychobiological model of exercise tolerance (6,7), the perceived exertion (referred to as “the conscious sensation of how hard, heavy, and strenuous a physical task is”; 8) plays a critical role: When the application of additional effort appears unreasonable or not possible, endurance performance (i.e., cycling) is terminated (6,7). Other frameworks like the taxonomy of fatigue argue that perceived fatigability (referred to as “the sensations that regulate the integrity of the performer based on the maintenance of homeostasis and the psychological state of the individual“; 9) is impacted by exercise-induced sensations like exertion or pain and thus influences performance fatigability (i.e., muscle and nervous system capacity to generate a sufficient response for a given exercise; 9). Exercise-induced pain itself is also an important determinant of endurance performance, as higher pain tolerance is associated with enhanced endurance performance (see 10 for a detailed overview). This is consistent with observations that former Olympic cyclists report successful coping with pain to be the greatest psychological challenge (11), and that being occupied with physical discomfort impaired performance (4). Corroborating the assumptions made in these theoretical frameworks, a focus group study with recreational endurance exercisers revealed that exercise-related sensations like exertion, pain, fatigue, or discomfort were the most common challenges (1). Beyond dealing with these bodily sensations, staying focused and motivated after difficult situations are further obstacles recreational exercisers report. However, sport-specific obstacles differ between athletes and non-athletes: For example, non-athletes are more likely to process physiological signals during sports negatively (e.g., sensations of exertion and pain) which might in turn trigger negative affective reactions (12). Also, non-athletes reported more pain, discomfort, or articulated irrelevant information for the exercise than athletes (13). Thus, lack of experience creates critical obstacles in sport.*  *Beyond focusing on perceived obstacles, it is also crucial to know which strategies exercisers spontaneously apply to deal with them. Deliberately implemented strategies and programs have been studied intensively (e.g., 2, 14) and show that endurance performance can be enhanced by mental imagery, self-talk, and goal setting (2). For instance, self-talk interventions can reduce perceptions of effort as one determinant of endurance performance (15). Active distraction at low training intensities was perceived as helpful in a sample of recreational runners to reduce boredom and increase positive emotions (16). Accordingly, metacognitive skills -pertaining to “planning and reviewing one’s attentional focus” (4), seem to be helpful for athletes in order to gain knowledge about the use of cognitive strategies. As with exercise-related obstacles, expertise and fitness level affect the kind of strategies exercisers use (17). Thus, a key characteristic of a strategy to enhance endurance performance is the adaptability to requirements and demand levels of different sports and athletes’ abilities. To our knowledge, research that elicits the specific obstacles exercisers face during a single bout of exercise, and that assesses the strategies exercisers use to cope with these challenges is lacking.* |
| **Issue 3**  2. Line 74: I agree that there it is a dearth of research that has specifically explored the mechanisms underpinning the potential success of an intervention in relation to endurance performance (i.e. see McCormick et al., 2015, for a discussion), but there are some think aloud studies that have explored obstacles (i.e. Whitehead, Jones, Williams, Dowling et al., 2019). Overall, I don’t think that the introduction has given justice to some of the research on endurance psychology that is out there. | Thank you for your comment. We made sure to incorporate additional literature into our overview of exercise-determining obstacles, also making a reference to the study by Whitehead et al. (2019, Reference #13). See our response to Issue 2 for details on how we edited this section. |
| **Issue 4**  3. An interesting point is raised in relation to the different physical and physiological demands of cycling (i.e. short prints and uphill cycling) and that these may require different psychological strategies. Most sports have different physiological demands (think of football as an example), so I would not think this is unique to cycling. Furthermore, although this may not have been tested directly in the cycling research, there are examples in the wider sport psychology literature. I would suggest to shift the focus on what evidence there is to suggest that tailoring one’s psychological strategy use to the specific demands of a task is helpful. This will help to show how this research builds on what is known in terms of the efficacy of interventions. | Thank you for your suggestion. We restructured this section and focused more generally on tailoring strategies to demands along the lines of your suggestion (p. 4-5).  *Exercise-induced obstacles and strategies to deal with them vary not only as a function of the exercisers’ training level and experience but also as a function of the task demands associated with an exercise. Within a given sport, physiological demands vary in terms of changes in neural blood circulations and metabolism (18), reduced oxygen distribution (19), and ionic and metabolic modifications in the concerned muscle (20). Consequently, these effects also affect our psychological response to an exercise. For instance, our affective response is modulated by the intensity of an exercise (21): At moderate intensities (i.e., up to the aerobic threshold) exerciser tend to have positive affective response, whereas at very high intensities (i.e., above the anaerobic threshold) the affective response tends to be negative. This difference in affective response has been explained (among others) by the different interoceptive sensations created by these intensities (21). The three-dimensional framework of perceived fatigability further highlights the differential contributions of sensory-discriminatory (i.e., perception of strain), affective-motivational (i.e., key emotion), and cognitive-evaluative components (i.e., mindset) to endurance performance (22). For example, a short anaerobic test can be completed by most exercisers, as it covers an exercising time of only 30 seconds, which might lead to a higher sense of achievability. However, the short duration of the test means that there is little time to adjust performance strategies. An aerobic test, on the other hand, is open ended and will therefore lead to complete exhaustion of the exerciser, likely leading to negative affective reactions. It is each participant’s decision when to terminate the test which also makes performance dependent on motivation and attitude. Due to the frequent increase in power output, the aerobic test probably requires significantly more self-control from the exerciser. Due to its longer duration, however, participants will also have more opportunities to adapt their strategies in order to last longer. Thus, different exercises (within one type of sport, as well as across sports) vary in the physiological and psychological demands they impose, which in turn might create obstacles that are very exercise-specific. To account for these differences, strategies need to be tailored to the obstacles an exerciser faces (23).* |
| **Issue 5**  4. A clear overview of the relevance of if-then planning is provided. From line 131-137: Could the authors specify what the effects of different types of goals are (i.e. process goals (keep going) or outcome/performance goals (time-based or based on outcome of event such as medals)? This is quite relevant considering the central role of goals in the study design. | Thank you for raising this interesting question. We agree that different types of goals could be relevant for endurance performance. Accordingly, we now address this issue in our discussion (p. 23-24).  *Finally, it is possible that the type of goals that participants set (e.g., process-related: keep going, ignore pain; outcome-related: target time) might have had an influence on endurance performance (see 86 about recommendations on effective goal setting in sports). We did not address this issue here because our focus was primarily on if-then planning rather than on goal setting. Still, future research should focus on how various types of exercise-related goals affect endurance performance.* |
| **Issue 6**  5. Line 150-151: To what extent do the author’s believe that self-efficacy is a key mechanism here? | Thank you for this question. Self-efficacy could indeed be a key mechanism when it comes to endurance performance. Accordingly,  we now discuss the potential relevance of self-efficacy (p. 24-25).  *In addition, there are a number of factors that potentially determine endurance performance but are beyond the scope of the present investigation. For example, future studies should take the influence of self-efficacy into account, a widely studied determinant of sports performance (e.g., 91) with beneficial effects on effort perception (92) and pain tolerance (93). Moreover, high self-efficacy is associated with improved implementation intention effects in complex tasks (94), suggesting that it might be crucial to ensure that participants feel confident with performing the athletic task to reap the potential benefits of forming if-then plans.* |
| **Issue 7**  6. The aims of the study are quite ambitious, and this has seemingly weakened the focus of the review of the literature and the research overall. Specific comments: Line 46: Suggest to replace marathon with ride to avoid confusion with running events. Line 49-50: Although there is a limited research on the psychological demands of endurance performance, I would be careful with the phrasing of that it is poorly understood. Line 57: What is meant with the limits of endurance performance? Clarification of this at the start of the paper would help to set the scene more clearly. | Thank you for your detailed feedback. We hope that by addressing your comments the focus of our literature review has improved. Moreover, we addressed your specific comments in the manuscript as follows (p. 3).  *The specific psychological obstacles that exercisers face during an endurance performance and the strategies they apply to deal with them requires more research as this topic is of great scientific and practical importance (1,2). To understand the limits of endurance performance (i.e., factors that hamper or disrupt endurance performance) and design effective sport psychological interventions, knowledge about the various exercise-related psychological obstacles that might affect performance is crucial (3).* |
| **Issue 8**  Method/Results: 1. A reference was made to statistical power – was a power analysis conducted? Ensure to include this. | We apologize for this omission. We have added the necessary information (p. 7):  *The sample size was determined in accordance with other implementation intention research in sports, following recommendations to enhance power in this type of research (e.g., employing a within-subject design; for a systematic review, see 33). A sample size of N = 54 participants is sufficient to detect a medium effect of f = 0.25 (Cohen, 1988) in a mixed factorial ANOVA to find a within-between interaction (α = .05) with a power of .95 (calculated with G*Power; 48).* |
| **Issue 9**  2. Seven participants dropped out, did the authors look at the data of these participants? Did those who dropped out still get course credit and the monetary compensation? There might be some insightful information here from the interviews as well. For example if they (participants who dropped out) all found it very hard and not enjoyable and there is a pattern, then this is worthwhile reporting. | Thank you for this helpful comment. Importantly, only three participants dropped out in the sense that they did not show up for Session 2. We looked at the data from these participants that were excluded and found no specific pattern that would explain why they dropped out. In order to clarify this aspect, we added the information to the manuscript about how many participants were excluded for which reason (p. 7):  *Participants were randomly assigned to conditions. Seven participants were excluded from quantitative data analysis because they did not participate in the second session (N = 3), due to technical difficulties (N = 3), or because they did not comply with instructions (N = 1).*  We also added the information regarding the participants’ compensation (p. 8):  *All participants signed an informed consent and were compensated with 30 Euro and course credit when they completed both study sessions.* |
| **Issue 10**  3. Participants: Would be insightful to have a greater idea of the sport activities the participants normally do – cycling was not anyone’s primary sport, yet on average they cycled a few hours a week, so it is a sport they are familiar with? How can they then be classified as exercisers with little or no prior cycling experience? I am also a bit confused about the hours per week that are reported, it is stated that the average exercise endurance training per week was 2.3hrs, which is the same as the number reported for cycling, but the SD is different? What happened here? Also, some greater clarity about the inclusion criteria would be useful. | We differentiated between riding a bike (e.g., for commuting to work) and cycling as a sport activity in our questionnaire. This means that participants might ride their bike during the week to get to university, for example, which would not necessarily mean that they are accustomed to the demands of a cycling training or that this number corresponds to the amount of time participants stated to perform an endurance sport. We clarified this and the other aspects in the manuscript as follows (p. 7):  *They reported to exercise M = 5.4 hours per week ± 2.9 (endurance training: M = 2.2 ± 2.2; strength training: M = 2.4 ± 2.2) and to be engaged in a variety of sport activities (most frequently reported were jogging 21.1 %, fitness 9.6 %, soccer, weight training 7.7 %), having performed their main sport activity for an average of M = 7.3 years ± 7.0. No participant reported cycling as their primary sport. Overall, participants stated to ride a bike for M = 2.3 hours per week ± 2 as a physical activity (e.g., to commute to work), and to spend M = 0.3 ± 0.7 hours per week with cycling as a sports activity. Participants in the goal and the implementation intention condition did not differ in their weekly training duration, p = .370, or the duration of performing their main sport, p = .340. There were no specific exclusion criteria for participation in the study, except that participants should have no injuries that prevented them from cycling.* |
| **Issue 11**  4. As an observation: The performance goal examples were not very specific – i.e. go for as fast as possible, or hold it for as long as possible. If they have not already done so, the authors may want to consider looking at some of the ‘do your best’ goal research (see Burton and colleagues) and the impact of the specificity of goals. If goals are vague, then it can be more difficult to commit to these, and this is something they can return to in the analysis of the if-then plans used in the results. | Thank you for pointing out this important point. Research on implementation intention research commonly resorts to this kind of goal (see Gollwitzer & Sheeran, 2006) because the focus is on planning effects. That said, it would be interesting to test whether the kind of goal (specific/unspecific) interacts with the effectiveness of implementation intentions. For instance, it might be that specific goals already improve performance and diminish the effects of if-then plans. To the best of our knowledge, this question has not yet been addressed so far. We added our thoughts about this issue to the discussion (p. 24):  *Also, future studies should consider the possible interaction of (un-)specificity of goals and if-then planning, as studies indicate that specific goals lead to better performance than vague, general or no goals (87) and goal specificity alone trumps the effects of merely ‘do your best’ goals (88). It is plausible that forming specific goals already improve endurance performance and thereby reduce implementation intention effects.* |
| **Issue 12**  5. Line 229: What was the justification of measuring RPE immediately before the test and what was the average at this stage? How quick was RPE taken after the test, and what were the instructions? | We added the missing information to the manuscript in the procedure section (p. 8):  *To assess perceived exertion, participants reported their RPE on the category ratio 10 (CR10) scale (47,49) prior to the test (to familiarize themselves with the use of the scale) and immediately after they completed it (a continuous measurement was not possible due to cycling at maximum power output).* |
| **Issue 13**  6. There are some issues with the conditions in terms that the participants in the goal intention condition were asked to reflect on the situations, thoughts, and actions they had stated in the structured interview. This could have (unintentionally) led to them setting spontaneous if-then plans and reflect on how they could do better. Did the authors consider this, and if so, how was this accounted for in the data analysis? Another issue is that the examples of ifs are quite vague or not necessarily a critical situation, i.e. if I have some power left. | Thank you for raising this important issue. Comparing an implementation intentions condition to a goal intention condition is a standard approach in if-then planning literature (Gollwitzer & Sheeran 2006) and effects are commonly observed even though participants in the goal intention condition might form spontaneous if-then plans. Still, it might be that small effect of the implementation intention are masked by spontaneous if-then planning. We mention this potential limitation in the discussion (p. 23):  *A closer look on our qualitative findings provides additional insights into the question of why if-then plans might have been ineffective in our study. In both tests, participants were able to identify obstacles and spontaneously applied several strategies that have shown to be useful by prior research (e.g., emotional regulation, attention regulation). This alone might have helped participants in the goal intention condition to improve their performance. Tentative support for this reasoning comes from the observation that participants substantially improved their performance in the aerobic test between the first and the second session, irrespective of whether they belonged to the goal or the implementation intention condition. Thus, participants might have been very effective in finding ways to deal with the challenges posed by the tests, making it difficult for the if-then planning intervention to further improve performance. Similar effects have been observed in a study investigating the influence of self-talk on performance in a time-to-exhaustion ergometer test, in which the expected effects of self-talk were masked by learning effects through task repetition and concomitant reflection of participants (84). Subsequent studies might resort to a less rigorous control condition in which participants do not engage in interviews about obstacles and strategies. Nevertheless, comparing an implementation intention condition with a goal intention condition is a standard approach in if-then planning research (34) and differences between conditions are commonly observed despite the possibility that participants in the goal condition might form spontaneous if-then plans.*  We agree with your argument that the if-then plans  specified by our participants might not have been  sufficiently specific. However, note that a critical  situation can refer to an opportunity, such as  having some power left (i.e., a critical situation is  not necessarily an obstacle; Bieleke &  Keller,2020). Moreover, we deliberately chose to  allow participants to make their own if-then plans  (Bieleke et al., 2020). In order to address these  ideas properly, we expanded the section on study  limitations that now reads as follows (p. 22-23):  *It is conceivable that participants in our study also planned behaviors that they thought would help them perform better, while in fact these behaviors might have been ineffective or even detrimental in some cases. In addition, critical situations were sometimes specified in vague terms (e.g., “If it becomes exhausting”) or did not pertain to an obstacle or opportunity (e.g., “If I am cycling”). This might be due to a lack of experience with cycling training, which required participants to base the specified obstacles and strategies on a single test performance. Consequently, it might be that if-then planning is a more effective strategy among experienced athletes (see 33, for a similar argument).* |
| **Issue 14**  7. Line 248: What does the literature say in terms of an ‘optimum’ number of if-then plans? If someone sets as many if-then plans is that a good thing? Would be useful to clarify this for the reader. | This is both an intriguing and a difficult question. For instance, making several plans for the same situation (i.e., a “Plan B”) might cause interference between plans and thus reduce their effectiveness (Vinkers et al., 2015). On the other hand, making several plans for different situations seems to be less problematic. We now discuss this important aspect (p. 23):  *Furthermore, plan effects in future studies might be enhanced by asking subjects to make one plan pertaining to the most critical obstacle (or opportunity) and the best goal-directed strategy. Rather than helping subjects prepare for different stressors in the cycling tasks, there might have been interference between the different if-then plans (85). Indeed, the majority of subjects in the implementation intention condition used more than one plan to prepare for the tests, which might have thwarted the beneficial effects of if-then planning.* |
| **Issue 15**  8. Line 264: Out if interest, by showing the participants feedback such as the time until the next power increase and so on, this can be a factor that can influence one’s coping. For example, if you know it is only a few seconds until the next step, then you keep going. They may have set themselves specific goals that influenced this. Is this something that was explored in the interviews and the data analysis? | Thank you for this interesting comment. Of course it is possible that the information on the screen might have influenced participants’ behavior and the statements evaluated in thematic analyses show that participants also used information on the screen to for instance set specific goals or to maintain an orientation over their performance. Importantly, even if participants specified goals pertaining to this kind of information it would not have changed our hypotheses about differences between conditions. |
| **Issue 16**  9. Line 303-304: How was this re-evaluation done and the accuracy assessed? Is this feasible and does this align with the principles of conducting thematic analysis. Interrater reliability is generally not advocated as part of thematic analysis and is surrounded with controversy. Having said this, it may work for the current study, but perhaps better as a content analysis, do consider the suggestions by O’Connor & Joffe (2020) for example: https://journals.sagepub.com/doi/full/10.1177/1609406919899220 A major issue with this type of coding is that one participant may have been quite in depth in their answers and as such they could have given multiple references to (as an example) motivation or frustration in their answers. What seems to have happened in the analysis (but I may have misunderstood) is that this was not accounted for, and as such the use of percentages in table S2x is meaningless. | Thank you for recommending this insightful article. We assessed interrater reliability to ensure a more objective understanding of the coding but report the results in detail only in the appendix for interested readers (see Table S1). We can also completely remove this information if you consider it unfitting.  In organizing the answers into codable items, we differentiated between thoughts, behaviors, and emotions. In the final analysis, we summarized the results into obstacles, strategies used, and potential strategies. So we agree with you that the mere frequencies of statements do not account for the fact how often or how excessively one participant mentioned a specific aspect (e.g., exertion). To address your concerns, we’ve decided to omit the frequency tables and now focus on categories, definitions and statements (see S2A – S2C Tables). This approach is also more in line with a qualitative approach to data analysis. |
| **Issue 17**  10. Looking at the data, it seems that missing drive and motivation was a major obstacle for many of the participants. This may well have affected a lot of the results and the effectiveness of the plans. | We absolutely agree with your assessment. We made sure to include this point in the discussion (p. 24).  *Another reason for the lack of efficacy of our intervention could pertain to the characteristics of the sample. None of our participants performed cycling as their main sport, meaning that they had little to no experience with the specific task demands. Research indicates that there could be differences between participants who regularly engage in a certain activity compared to inexperienced participants in terms of their motivation to perform well (89). With regard to sport psychological interventions, it has been argued that inexperienced samples lack the intrinsic motivation to perform the task, therefore obscuring any effects of self-regulatory strategies that are based on sufficient motivation (3). The results of our qualitative analysis mirror this argument, as many participants reported missing drive and motivation as obstacles for performing well. However, our participants reported to be well committed in both conditions. Nevertheless, these results should be interpreted with care because they might reflect social desirable responding.* |
| **Issue 18**  11. A nice insight was provided into the content of the if-then plans, and this is valuable data to share. | We are grateful that you like this aspect of our work. |
| **Issue 19**  12. Perhaps unfortunately, there seemed to be a difference in terms of power for both tests between the two conditions at the first session which is difficult to control for at the start. Nevertheless it is interesting that, for the aerobic test the time to failure between the two conditions was pretty much identical. Perhaps this tells us more about the appropriateness of the test, rather than the effectiveness of if-then plans. A discussion of this and whether a 20 minute time-trial (for example) would have been more suitable. | Indeed, baseline differences make the analysis of performance more intricate. The higher mean power in the implementation intention condition might have made further increases difficult, masking potentially beneficial effects of the intervention. We now discuss this issue, along with alternatives to the aerobic test we used (p. 25).  *In the same vein, it can be discussed whether a different performance test could have been more appropriate to show effects of if-then plans. As the aerobic test requires a frequent increase in power output and thus poses increasing self-regulatory demands, a test that feels more achievable (e.g., a time trial) could have heightened participants’ perceptions of self-efficacy. However, as a self-paced time trial would induce further self-regulatory demands (e.g., constantly having to adjust one’s power output to what one believes one can sustain for the expected ride time), this would be a less pure measure of aerobic performance – as pacing errors can substantially affect overall performance - compared to the aerobic test. A difference in mean power between conditions in Session 1 makes the elicitation of if-then planning effects even more intricate. On the one hand, as participants in the implementation intention condition already presented higher power output, additional enhancement of performance might have been difficult which could obscure potential if-then planning effects. On the other hand, performance parameters do not indicate that further improvement through if-then planning is not possible, which again raises the question of the appropriateness of the tests to illustrate performance enhancements. Future studies should investigate which tests are optimally suited to reflect if-then planning effects on endurance performance.* |
| **Issue 20**  Discussion: A few comments that I have made earlier could be considered in the discussion, such as the appropriateness of the cycling task used (for example, why not a self-paced task, or a 20 min time trial), drop out of participants, the alternative condition (goal condition) chosen, discussing of mechanisms that can make if-then plans effective (perhaps self-efficacy) that could have been measured? A combination of the latter two points is relevant as the type of tasks (regularly used as indicators of fitness and peak power) may have prevented the authors to identify a ‘performance’ change but the participants may have felt more confident. I am not surprised that there was no effect on performance. Thus, I would not necessarily support the conclusion that there is a lack of effectiveness of the if-then plans, because effectiveness was not actually considered beyond a performance measure of power (arguably this focused more on efficacy than effectiveness? See Bishop, 2008 for a discussion of effectiveness and efficacy). | Thank you for these suggestions. We added these ideas to the discussion (see our responses to your previous comments) and feel that the manuscript has benefitted substantially from these changes. In regard to the discussion of Bishop (2008), we changed the wording from effectiveness to efficacy, as this captures the aim of researching if-then planning in endurance at this point more clearly. |
| **Issue 21**  A discussion around the conditions used and the potential for participants in both conditions to have used if-then plans is needed. A more passive control condition could have strengthened this study. | Thank you for your feedback. We expanded the discussion on that aspect (p. 23):  *Subsequent studies might resort to a less rigorous control condition in which participants do not engage in interviews about obstacles and strategies. Nevertheless, comparing an implementation intention condition with a goal intention condition is a standard approach in if-then planning research (34) and differences between conditions are commonly observed despite the possibility that participants in the goal condition might form spontaneous if-then plans.* |
| **Issue 22**  What do the authors feel the implications are of conducting this type of research with participants who are not involved or invested in the task? For example, in this sample the participants were not involved in cycling – how do we know that they invested in the task and thus felt that using an if-then plan would be meaningful for them? There is some insightful discussion around this in the limitations, and I wonder whether the researchers could consider how to address this in the analysis? | Thank you for this important thought, which is related to Issue 17 (see above). We took the issue of motivation into account by assessing goal commitment and comparing it between conditions. As we have outlined above, these results suggest a sufficient level of motivation; however, these might be biased by social desirability. There was also no difference between conditions, indicating that it does not affect our results. Please see our response to Comment 17 for details regarding how we addressed this issue in the discussion. |

**Our response to the comments of reviewer 2:**

| **Reviewers’ comments** | **Our response** |
| --- | --- |
| **General comments:** The topic of the present manuscript is of interest, because it investigates ‘psychological’ aspects of endurance performance, which importance has become increasingly clear over the last years. However, from my point of view, there are major issues that should be addressed before this manuscript can be considered for publication in Plos One. These points include the following (see specific comments for details). (i) In general, the manuscript is too long and uses many words for aspects that should be described more briefly and straight forward. Moreover, plain language is used in some places, which should be avoided. (ii) The introduction is too long and mixes up general information and detailed study-related information, which in part are better suited in the methods section. Furthermore, the general determining factors of endurance performance should be mentioned to derive the research question. In this context, mechanistic foundations for why exercise-induced obstacles should differ between tasks should be presented. (iii) Performing both tests in one session introduces the problem of ‘residual fatigue’, which results in a potential bias of the parameters recorded during the second test induced by ‘history-dependent’ modulations. (iv) The methods lack some information for specific measures, which should be clarified. (v) The discussion is not appropriate from my point of view and should be amended. The main points are the same as for the introduction. | Thank you for your detailed feedback on our manuscript. We feel that it has substantially benefitted from your suggestions. We hope that you like how we implemented your feedback.   (i) We shortened the manuscript in general and adopted your suggestions, for instance in the results section (Issue 23, S2A-S2C Tables). (ii) Also, the introduction has been revisioned and does now describe our research approach more consisely. Method-related information has been repositioned in the method section (Issue 9). We made sure to implement the determining factors of endurance performance (e.g., effort, pain, fatigue; Issue 2) as a foundation for our research question. We gratefully accepted your proposal to present the mechanistic foundations of exercise-related obstacles (Issue 4 and 5). (iii) Thank you for raising this important limitation. We agree with your evaluation that this influences performance in the second test and addressed this concern specifically in our discussion (Issue 15). (iv) We apologize for these inclarities and made sure to implement the necessary information (Issue 10, Issue 11, Issue 12, Issue 13, Issue 17 and Issue 19). (v) We agree with your observation that some aspects could be more thoroughly discussed. This is why we expanded the discussion incorporating your ideas regarding biased parameters in the second test, sample heterogeneity and specification (Issue 15, Issue 21, Issue 24-29). |
| **Issue 1**  **1. Introduction:** l45-55: From my point of view, presenting the short everyday example and the aims of the study at this point is not optimal and brings no advantage compared to the “standard” introduction, which tries to summarize the studies related to the topic and present the aims of the study afterwards. Therefore, I recommend to restructure the introduction accordingly. l60: Please insert a name before reference (2) to optimize the sentence. l64: Please insert a name before reference (5) to optimize the sentence. l69: Please insert a name before reference (7) to optimize the sentence. Please check the correctness of sentences, when you change the citations style. | Thank you for your recommendation. We restructured the introduction along the lines of your suggestions. It now reads as follows (p 3):  *The specific psychological obstacles that exercisers face during an endurance performance and the strategies they apply to deal with them requires more research as this topic is of great scientific and practical importance (1,2). To understand the limits of endurance performance (i.e., factors that hamper or disrupt endurance performance) and design effective sport psychological interventions, knowledge about the various exercise-related psychological obstacles that might affect performance is crucial (3). Accordingly, a growing body of research focuses on the obstacles that endurance athletes have to deal with during performance (e.g., 1,4) and comprehensive reviews shed light on the characteristics of psychological interventions in sport that allow them to improve endurance performance (e.g., 5).*  Thank you for your corrections. We changed the style in all instances. Please excuse these errors. |
| **Issue 2**  l57-74: From my point of view, the determinates of endurance performance should be introduced at this point, with a focus on ‘perceptual and/or ‘psychological’ aspects. For example, we know, e.g., from the Marcora and Mauger papers, that effort as well as exercise-induced pain perception are key-determinates of endurance performance in humans. It seems obvious, that at least these things should be also self-reported obstacles. After introducing the ‘psychological’ key-determinates of endurance performance, it makes sense to present the data regarding the perceived obstacles of athletes and physically inactive people. Moreover, since endurance performance is, at the end, limited by state fatigue mechanisms, the taxonomy of fatigue provided by Enoka and Duchateau (2016) could also be a theoretical foundation for your experiment. Within this framework perceived fatigability is determined by, e.g. exercise-induced sensations. | Thank you for your detailed and helpful suggestions. We now cover these theoretical approaches in our discussion, outlining how they inform our present research. We incorporated the suggested publications by Marcora (2008(; Reference #6), Marcora et al. (2008; #7), Enoka & Duchateau (2016; #9) and by Mauger (2019; #10). The corresponding section now reads as follows (p. 3):  *According to the psychobiological model of exercise tolerance (6,7), the perceived exertion (referred to as “the conscious sensation of how hard, heavy, and strenuous a physical task is”; 8) plays a critical role: When the application of additional effort appears unreasonable or not possible, endurance performance (i.e., cycling) is terminated (6,7). Other frameworks like the taxonomy of fatigue argue that perceived fatigability (referred to as “**the sensations that regulate the integrity of the performer based on the maintenance of homeostasis and the psychological state of the individual“; 9) is impacted by exercise-induced sensations like exertion or pain and thus influences performance fatigability (i.e., muscle and nervous system capacity to generate a sufficient response for a given exercise; 9). Exercise-induced pain itself is also an important determinant of endurance performance, as higher pain tolerance is associated with enhanced endurance performance (see 10 for a detailed overview). This is consistent with observations that former Olympic cyclists report successful coping with pain to be the greatest psychological challenge (11), and that being occupied with physical discomfort impaired performance (4). Corroborating the assumptions made in these theoretical frameworks, a focus group study with recreational endurance exercisers revealed that exercise-related sensations like exertion, pain, fatigue, or discomfort were the most common challenges (1). Beyond dealing with these bodily sensations, staying focused and motivated after difficult situations are further obstacles recreational exercisers report. However, sport-specific obstacles differ between athletes and non-athletes: For example, non-athletes are more likely to process physiological signals during sports negatively (e.g., sensations of exertion and pain) which might in turn trigger negative affective reactions (12). Also, non-athletes reported more pain, discomfort, or articulated irrelevant information for the exercise than athletes (13). Thus, lack of experience creates critical obstacles in sport.* |
| **Issue 3**  l79-81: It is not purposeful for me to present the aims of the study after every subheading. Therefore, please concentrate of the key-elements of your study and present them straight forward in a short introduction, which ends with the aims of the study. | Thank you, we have restructured this part of the paper according to your recommendations and presented the aims of the study at the end of the introduction (p. 6).  *Taken together, the first aim of this study is to use a qualitative approach to investigate 1) the specific obstacles exercisers face during two different exercises, 2) the strategies they use spontaneously to deal with these obstacles, and 3) the strategies they consider helpful for future performances of the test. These obstacles and strategies were assessed after a first session with an anaerobic (Wingate test) and an aerobic cycling test (incremental exercise test). The second goal is to investigate whether if-then plans can help exercisers to better deal with the obstacles they face during an anaerobic and an aerobic endurance test. To this end, we used the obstacles and strategies that participants reported in the first session to help them generate individually tailored if-then plans for a second session with the same two tests. In line with previous research (44,45), we expected improved performance in the if-then plan condition compared to a goal intention condition. As control variables, we assessed ratings of perceived exertion (RPE; 47) and goal commitment in both sessions.* |
| **Issue 4**  l82-88: You have mixed study characteristics with the general presenttion of facts. This is not meaningful to me. At this point you would like to say that the type of obstacle as well as the coping strategy depend on the exercise-intensity, irrespective of your concrete task, i.e. cycling. This is very plausible, since the physiological requirements are different leading, e.g., to higher effort and exercise-induced pain perception during exercise, which can be attributed to (neuro)physiological mechanisms. Therefore, I recommend to state the fact that obstacles as well as strategies might depend on exercise intensity. Thereafter, you should state why this is the case, e.g. metabolic requirements resulting in an increased intensity of different perceptual qualities and a decrease in affective valence (see Venhorst and Ekkekakis papers for these aspects). | Thank you very much for your input. We have changed the section in the paper to adopt your argumentation. We incorporated the suggested publications by Ekkekakis (2003; Reference #21) and by Venhorst et al. (2018; Reference #22). It now reads as follows (p. 4-5):  *Exercise-induced obstacles and strategies to deal with them vary not only as a function of the exercisers’ training level and experience but also as a function of the task demands associated with an exercise. Within a given sport, physiological demands vary in terms of changes in neural blood circulations and metabolism (18), reduced oxygen distribution (19), and ionic and metabolic modifications in the concerned muscle (20). Consequently, these effects also affect our psychological response to an exercise. For instance, our affective response is modulated by the intensity of an exercise (21): At moderate intensities (i.e., up to the aerobic threshold) exerciser tend to have positive affective response, whereas at very high intensities (i.e., above the anaerobic threshold) the affective response tends to be negative. This difference in affective response has been explained (among others) by the different interoceptive sensations created by these intensities (21). The three-dimensional framework of perceived fatigability further highlights the differential contributions of sensory-discriminatory (i.e., perception of strain), affective-motivational (i.e., key emotion), and cognitive-evaluative components (i.e., mindset) to endurance performance (22). For example, a short anaerobic test can be completed by most exercisers, as it covers an exercising time of only 30 seconds, which might lead to a higher sense of achievability. However, the short duration of the test means that there is little time to adjust performance strategies. An aerobic test, on the other hand, is open ended and will therefore lead to complete exhaustion of the exerciser, likely leading to negative affective reactions. It is each participant’s decision when to terminate the test which also makes performance dependent on motivation and attitude. Due to the frequent increase in power output, the aerobic test probably requires significantly more self-control from the exerciser. Due to its longer duration, however, participants will also have more opportunities to adapt their strategies in order to last longer. Thus, different exercises (within one type of sport, as well as across sports) vary in the physiological and psychological demands they impose, which in turn might create obstacles that are very exercise-specific. To account for these differences, strategies need to be tailored to the obstacles an exerciser faces (23).* |
| **Issue 5**  l91-100: The same issue as before. You present a lot of information about your study in the introduction. However, this is not meaningful for the readability and understanding of your study. Therefore, I suggest, as mentioned above, that different exercises have, e.g., different metabolic requirements (anaerobic vs. aerobic). Which test and task you have used is not relevant at this point and should be mentioned first in the aims of your study at the end of the discussion and described in detail in the methods. l96: This is not relevant and in addition not the gold standard to prescribe training intensities. | Thank you for your helpful comment, we restructured this part of the paper entirely (see Issue 4). |
| **Issue 6**  101-113: This is a general description of the test and its characteristics. You infer that tests would differ regarding the obstacles without a (neuro)physiological description why this should be the case. This is a general limitation of the present manuscript as it is very descriptive and without a mechanistic basis, e.g., for effort and pain perception. | Thank you for this important feedback. See our response to Issue 4 for details on how we edited this section. |
| **Issue 7**  l114-155: This part reads better and adopts a global perspective. However, it is far too long and should be shortened. | Thank you for this observation. We shortened this part considerably and it now reads as follows (p. 5-6):  *Optimal performance hinges on effective self-regulation, the importance of which for sports performance is well established (e.g., 24). Beyond relying on their intuitive lay knowledge, exercisers might benefit from employing targeted self-regulatory strategies to deal more effectively with their exercise-induced obstacles. One promising self-regulatory (25) strategy is if-then planning (often referred to as implementation intentions; 26). Implementation intentions are an effective self-regulation strategy in a variety of contexts (27,28), like in the context of physical exercise (29), health (e.g., 30–32), and researchers have started to investigate its effects on endurance performance (e.g., 33). If-then planning is based on establishing links between goal-relevant situations (e.g., obstacle: experiencing the urge to stop) and goal-directed behaviors (e.g., overcoming the obstacle: cheering yourself on) in an if-then format: “If I encounter obstacle X, then I will perform behavior Y!” (e.g., “And if I feel the urge to stop, then I tell myself: You can do it!”; 34). The structure of if-then plans is assumed to facilitate goal attainment by strengthening the mental representation of the goal-relevant situation, making it more accessible and easier to recognize (26,35). Moreover, if-then planning is assumed to automate the initiation of the goal-directed behavior (36).*  *If-then planning is typically contrasted with forming goal intentions, which refers to merely specifying a desired outcome or a desired behavior (e.g., “I want to keep going as long as possible!”; 37). Because such goals comprise no specific link between a situation and a behavior, they are conducive to more deliberative, top-down ways of self-regulation than if-then planning, which facilitates automatic, bottom-up ways of self-regulation. Importantly, research shows that when it comes to staying on track, even when the performing the goal-directed behavior is perceived as aversive, if-then planning is a more effective self-regulation strategy than goal setting (38). Moreover, if-then plans are effective for counteracting impulsive reactions (39). For example, when an athlete has to control the impulse to follow every acceleration of her opponents (because not controlling this impulse would eventually wear out the athlete, reducing the chance to win the race). Their desirable cognitive mechanisms (e.g., automaticity) and their effectiveness across various domains (e.g., health, exercise) render if-then plans a promising self-regulatory strategy in endurance sports (40). However, prior research in this domain has so far produced mixed results (see 33 for a comprehensive overview): plans focusing on one or two obstacles defined by the experimenter have been investigated (41–43) and observed inconsistent results. Bieleke et al. (33) argue that the effectiveness of if-then plans might be enhanced by tailoring if-then plans to athletes – that is, instructing exercisers to generate their own plan contents geared towards their personal obstacles and strategies to deal with them. For example, studies in which if-then plans were used without providing a particular obstacle in non-endurance domains (e.g., tennis, 44; or golf and darts, 45) found positive effects on performance. Thus, the heterogeneity of prior findings might be due to a lack of adequately tailoring if-then plans to the challenges an exerciser faces during the exercise (46).* |
| **Issue 8**  l156-165: Here again you present the next aim of the study. As I wrote before, writing a manuscript in this way is not meaningful for me and leads to less understanding. However, I am open for arguments. | As presented in Issue 3, we restructured the introduction according to your suggestions and presented the aim of our study at the end of the introduction. |
| **Issue 9**  l167-189: This part is also too detailed for an introduction. It reads like a ‘study procedure’ section, which should be placed in the methods. Although I prefer to give a short study overview in the introduction, I recommend to shorten this paragraph. | Thank you for your recommendation. We shortened this paragraph significantly and feel that it explains the aim of the study now more clearly. We hope that you like how we implemented your ideas (p. 6).  *Taken together, the first aim of this study is to use a qualitative approach to investigate 1) the specific obstacles exercisers face during two different exercises, 2) the strategies they use spontaneously to deal with these obstacles, and 3) the strategies they consider helpful for future performances of the test. These obstacles and strategies were assessed after a first session with an anaerobic (Wingate test) and an aerobic cycling test (incremental exercise test). The second goal is to investigate whether if-then plans can help exercisers to better deal with the obstacles they face during an anaerobic and an aerobic endurance test. To this end, we used the obstacles and strategies that participants reported in the first session to help them generate individually tailored if-then plans for a second session with the same two tests. In line with previous research (44,45), we expected improved performance in the if-then plan condition compared to a goal intention condition. As control variables, we assessed ratings of perceived exertion (RPE; 47) and goal commitment in both sessions.* |
| **Issue 10**  2. Methods l193: Have you performed a sample size calculation based on the effect sizes of previous studies? | We apologize for omitting this information. We have added the necessary information (p. 7):  *The sample size was determined in accordance with other implementation intention research in sports, following recommendations to enhance power in this type of research (e.g., employing a within-subject design; for a systematic review, see 33). A sample size of N = 54 participants is sufficient to detect a medium effect of f = 0.25 (Cohen, 1988) in a mixed factorial ANOVA to find a within-between interaction (α = .05) with a power of .95 (calculated with G*Power; 48).* |
| **Issue 11**  l202: I suggest to present means plus/minus standard deviations, because it is easier to read. | Thank you for this suggestion. We now present this information as you suggested. |
| **Issue 12**  l218: Have you conducted the experiments on the same time of the day? | We added this information to the manuscript (p. 8).  *The second session took place 7 to 14 days after the first session at the same time as the first session (if possible for participants).* |
| **Issue 13**  l229: What do you mean with RPE assessment before and after the test? Effort can only perceived related to a task. Do you mean at the beginning and end of the task? Or retrospectively after the task? | We are sorry for this confusion. We clarified this part in the manuscript and it now reads as follows (p. 8):  *To assess perceived exertion, participants reported their RPE on the category ratio 10 (CR10) scale (47,49) prior to the test (to familiarize themselves with the use of the scale) and immediately after they completed it (a continuous measurement was not possible due to cycling at maximum power output).*  Additionally, we removed the values of start RPE from Table 2, as we agree that these values are not relevant for our analyses (p. 17). |
| **Issue 14**  l230: I think ‘elicit’ is not a good term in this regard. | Thank you for your correction, the wording has been changed. |
| **Issue 15**  l232-233: Why have you performed the aerobic test on the same day? It is known that perceptual and physiological process are ‘history-dependent’. Therefore, your results for the aerobic activity might be biased due to the prior anaerobic activity. Please comment on this in a limitation of the study section. | Thank you for this feedback. We agree with your observation that these processes possibly bias the performance parameters in the second test. We included this important aspect as a limitation in the discussion (p. 25).  *Additionally, conducting both performance tests on the same day is a limitation of this study from an exercise physiology perspective,: Perceptual and physiological processes in the first test might have influenced those in the second test (e.g., in terms of residual fatigue; see 95 for a detailed review).* |
| **Issue 16**  l269: I suggest to use ‘time-to-exhaustion’ because the term ‘time to failure’ is critically discussed. | Thank you for your suggestion. We have changed this wording throughout the manuscript. |
| **Issue 17**  l273: What do you mean with RPE assessment before and after the test? Effort can only perceived related to a task. Do you mean ta the beginning and end of the task? Or retrospectively after the task? | Thank you for pointing us to this issue. We have reworded the sentence. Please see our answer to Issue 13. |
| **Issue 18**  l325: Please insert a name before reference (47) to optimize the sentence. The same applies to l325. Please check and amend this aspect in the whole manuscript. I will not mark it again | We apologize for this error. The references have been corrected and sentences been checked for wording. |
| **Issue 19**  l322: Unfortunately, I am not familiar with the Bayesian t-test. For my understanding, you have a within group comparison for the tasks and afterwards you compare different groups with regard to the coping strategies. Can these dependent and independent measures be mixed up in one statistical model? | We apologize for this confusion. We have clarified our analyses as follows and hope that our approach is now clear (p. 12):  *First, we controlled if goal commitment in both tests and RPE finish in the anaerobic test differed between the goal intention and the implementation intention group and between sessions with a 2 (Condition: goal vs. implementation intention condition) × 2 (Session: 1 vs. 2) Bayesian mixed-factor ANOVA. For testing if RPE increased during the aerobic test and whether these increases differentiate between both conditions and sessions, we performed an analogous analysis with RPE as dependent variable, where we added Test duration as additional within-participants factor for the aerobic test (to account for differences in test duration in the aerobic test, RPE values were standardized from 0 to a 100 percent and then aggregated across 20 % intervals).*  *In addition, we checked for any performance differences between both groups in Session 1 conducting Bayesian t-tests, for which we report Bayes factors (BF10).* |
| **Issue 20**  3. Results l349: Please provide a legend for table 1 clarifying the meaning of ‘*’. | We are sorry for this omission. This information has now been added (p. 13).  *Note. * refers to obstacles / strategies that were only mentioned after the anaerobic test, while ** references obstacles / strategies that were only mentioned after the aerobic test.* |
| **Issue 21**  l359-361: Here you provide obstacles that might be specific to you recreational active participants. It might be that inactive populations describe other obstacles. These subjects-specific factors should be emphasized in the discussion and limitations of the study section. | Thank you for raining this very important issue. We included this limitation in the discussion (p. 26 -23).  *A generalization to more (e.g., athletes) or less (e.g., physically inactive) experienced populations can only be made to a limited extent. Future research should investigate the extent to which these findings hold in other populations as well.* |
| **Issue 22**  l355-391: For some answers you indicate the frequency using percentages but for others not. Please include this information consistently. | To address the comment of the other reviewer that these frequencies are difficult to interpret and in order to streamline the presentation of the analyses, we omitted this information (see S2A – S2C Tables). |
| **Issue 23**  l355-469: This part of the results section is very long. Is it perhaps useful to present the statements and frequencies in a table? | Thank you for this helpful suggestion, we transferred the corresponding information to our existing tables (see S2A – S2C Tables) and shortened the section in turn (p. 12 – 16). |
| **Issue 24**  Discussion l523: Since your participants were recreational active, I assume that everybody has cycling experience. I think what you mean is experience with cycling training or something like that. Please specify this aspect. | Thank you. This aspect has been clarified to define our sample more precisely (p. 19-20).  *We assessed the obstacles exercisers with little or no prior cycling training experience faced during two different whole-body endurance cycling tests, along with the strategies they spontaneously applied to deal with these obstacles and strategies they considered helpful for future performances of the tests.* |
| **Issue 25**  l523: The word ‘cardiovascular’ is not meaningful in this sentence. | Thank you for your correction. This instance has been corrected. |
| **Issue 26**  l543: You have to specify your sample. In the introduction you have justified your investigation with the argument that these aspects were often investigated in endurance athletes. Later in the manuscript you have stated that the majority of subjects had no or little experience with cycling. However, here you stated that 16 of them were endurance athletes. | We are very sorry for this inconsistency. We revised the manuscript accordingly and now refer to the sample in a coherent way (p. 20).  *Interestingly, while McCormick et al. (1) interviewed endurance athletes, only a small portion of our sample were active in endurance sports (n = 16) and even these athletes had negligible experience with cycling tests.* |
| **Issue 27**  l529-568: As for the introduction, you have to find the way back to a model or the determinates of endurance performance and have to discuss your results in the context of the known factors (e.g. effort and pain perception). This should be done similar to the discussion of boredom. This is a general weakness of your manuscript. You have some parts that are well written and structured, interspaced by paragraphs that are written in plain language and with too many words. | Thank you for your feedback. We made sure to discuss this aspect in the discussion and to reflect how the proposed determinants of endurance performance are reflected in our dataset (p. 20-21).  *The importance of perceived exertion as a major performance-limiting obstacle aligns well with predictions of the psychobiological model (6,7), supporting the crucial role perceived exertion plays for endurance performance. Furthermore, the relevance of reported exertion, pain, and discomfort seems to be characteristic of less experienced samples (e.g., 13) and it can be assumed that a majority of our sample was overwhelmed by the external demands of the task (e.g., riding on an ergometer, staying in a seated cycling position) and their physical challenges (e.g., heat stress in the laboratory, muscle pain) that their concentration on the performance test objectives was limited.* |
| **Issue 28**  l603: From my point of view, formulations like ‘grain of salt’ are not appropriate for scientific manuscripts. | Thank you, the wording has been changed. |
| **Issue 29**  l617: I think that the heterogeneity of participant’s characteristics is a major limitation. Irrespective of the concrete task (e.g. cycling and running), endurance athletes interpret their physiological signals differentially compared to nonathletes in the different intensity domains. This might have biased/consequences for your outcome. | We absolutely agree with your evaluation and made sure to include this limitation in our discussion (p. 21).  *Especially during the anaerobic test, technical aspects of cycling like acceleration, power management, and slowing down were reported as critical by participants, which converges with findings that less experienced athletes experience greater difficulty in regulating their efforts (69) and in dealing with their physiological sensations compared to more experienced athletes (13).* |
