# Supplementary material for: Struggles and strategies in anaerobic and aerobic cycling tests: A mixed-method approach with a focus on tailored self-regulation strategies
Source: PLoS One. 2021 Oct 27;16(10):e0259088. doi: 10.1371/journal.pone.0259088 (PMC8550367; doi:10.1371/journal.pone.0259088)
Supplement: Attachment — Submitted filename: Response to Reviewers.docx [file pone.0259088.s006.docx]

Universität Konstanz · Fach · 78457 Konstanz

Anna Hirsch

Wissenschaftliche Mitarbeiterin

Department Sportwissenschaft, Sportpsychologie

Universitätsstraße 10

78464 Konstanz

Anna.Hirsch@uni-konstanz.de

www.uni-konstanz.de

Universitätsstraße 10

78464 Konstanz

Deutschland

05.10.2021

Revision for: PONE-D-21-13120

*Dear Mr. Gruet,*

*thank you and the reviewers for the helpful reviews concerning our paper entitled “Struggles and Strategies in Anaerobic and Aerobic Cycling Tests: A Mixed-method Approach with a Focus on Tailored Self-Regulation Strategies" submitted to PLOS ONE (Manuscript ID: PONE-D-21-13120R1).*

*We addressed the final remarks raised by the reviewers and edited the manuscript accordingly (for a detailed answer to each issue that was raised, please see table below). The revised version of the manuscript is 37 pages long and includes 2 Figures and 4 Tables (additionally, 4 Tables and 1 Figure in the Supporting information).*

*We again thank you for your constructive review. We hope for further positive feedback from you, and we would be honored if our paper would be accepted for publication in PLOS ONE.*

*Sincerely,*

*Anna Hirsch, Maik Bieleke, Raphael Bertschinger, Julia Schüler & Wanja Wolff*

| **Journal Requirements:** | **Our response** |
| --- | --- |
| **Issue 1**   Please review your reference list to ensure that it is complete and correct. If you have cited papers that have been retracted, please include the rationale for doing so in the manuscript text, or remove these references and replace them with relevant current references. Any changes to the reference list should be mentioned in the rebuttal letter that accompanies your revised manuscript. If you need to cite a retracted article, indicate the article’s retracted status in the References list and also include a citation and full reference for the retraction notice. | Thank you for your feedback on our manuscript. We made sure to not use retracted papers and especially checked the preprints that we cited (e.g., Wolff et al., 2021, which has been published in the meantime). We assumed that the cited preprints raised the question about retracted papers. If it is another paper that we have not been able to identify so far, please let us know.  According to the reviewers’ feedback, we added the following references to the manuscript:  # 25 Baumeister RF, Vohs KD. Self-regulation, ego depletion, and motivation. Social and Personality Psychology Compass 2007;1(1):115–28. doi: 10.1111/j.1751-9004.2007.00001.x.  #54 Paquette M, Le Blanc O, Lucas SJE, Thibault G, Bailey DM, Brassard P. Effects of submaximal and supramaximal interval training on determinants of endurance performance in endurance athletes. Scand J Med Sci Sports 2017;27(3):318–26. doi: 10.1111/sms.12660. PubMed PMID: 26887354.  #55 Hofman N, Orie J, Hoozemans MJM, Foster C, Koning JJ de. Wingate Test as a Strong Predictor of 1500-m Performance in Elite Speed Skaters. Int J Sports Physiol Perform 2017;12(10):1288–92. doi: 10.1123/ijspp.2016-0427. PubMed PMID: 28253027. |

**Our response to the comments of reviewer 1:**

| **Reviewers’ comments** | **Our response** |
| --- | --- |
| **General comments:**  Well done to the authors for putting in the effort to addressing the extensive range of comments of both reviewers. The key messages of the study come across a lot more clearly now. I have made a few more comments – the line numbers represent the line numbers of the ‘track changes’ document from page 51 of the full pdf document onwards. | Thank you very much for the positive reception of our revised manuscript and for the further helpful comments you made on the edited version. We feel that the manuscript benefitted again from your review and we hope that you like how we implemented your feedback. |
| **Issue 1**   Line 111 – rephrase, ‘to an exercise’ does not read well | Thank you for your feedback. We rephrased this sentence and it now reads as follows (p. 4):  *Consequently, these effects also affect our psychological response to athletic activities.* |
| **Issue 2**  Line 123-124 – Is there a supporting reference that can be cited here? | Thank you for your helpful suggestion. We have added a supporting reference (#23) and the sentence now reads as follows (p. 5):  *Due to the steady increase in power output, the aerobic test probably requires significantly more self-control from the exerciser (see (23) for a similar reasoning). Due to its longer duration, however, participants might also have more opportunities to adapt their strategies in order to last longer.* |
| **Issue 3**  Line 199 – Ensure to provide definition of self-regulation, so that the reader is familiar with the authors’ interpretation of self-regulation | Thank you for this important suggestion. Indeed, this information is lacking at this section of the manuscript and would be beneficial for the reader. We have added the definition of Baumeister and Vohs, 2007 (#25) and the sentence now reads as follows (p. 5):  *Optimal performance hinges on effective self-regulation (i.e., “the capacity of organisms (here, human beings) to override and alter their responses” (25)), the importance of which for sports performance is well established (e.g., (26)).* |
| **Issue 4**  Line 216- Remove ‘the’ before ‘performing’ | Thank you for your correction, this error has been removed. |
| **Issue 5**  Line 218-220 – Sentence does not read well | Thank you for your feedback, we have changed this passage and it now reads as follows (p. 6):  *Moreover, if-then plans are effective for counteracting impulsive reactions (40), which can be helpful for example when an athlete has to control the impulse to follow every acceleration of her opponents (because not controlling this impulse would eventually wear out the athlete, reducing the chance to win the race).* |
| **Issue 6**  Line 380-386: Can this be rephrased for clarity | Thank you very much for this correction. Indeed, the sentence structure led to ambiguity. After editing, the passage reads as follows (p. 8-9):  *To assess perceived exertion, participants reported their RPE on the category ratio 10 (CR10) scale (48,50) prior to the test (to familiarize themselves with the use of the scale) and immediately after they completed it (a continuous measurement during the anaerobic test was not possible due to cycling at maximum power output). In the following fifteen-minute break, goal commitment was assessed, and a structured interview was conducted which explored perceived obstacles of participants, the strategies they spontaneously employed to deal with these obstacles, and the strategies they deemed helpful in future performances of the test (see study materials in S1 Appendix).* |
| **Issue 7**  Line 521-522: Would suggest to remove interrater reliability and the range of agreement as outlined in my first review. The focus can then shift to the discussion of agreeing to the themes. | Thank you for your valuable suggestion. We have removed the interrater reliability accordingly (p. 12). |
| **Issue 8**  Line 560 – Data are plural, change to were For the thematic analysis – normally the reader would expect to see some direct quotes to illustrate the themes – if there are any particular quotes that can bring a theme come to ‘life’ then I would suggest that the authors bring some (not all) of these back into this section. | *Thank you for your correction, this error has been corrected.*  As for the thematic analysis, we agree with your assessment that some quotes to illustrate the themes would be helpful for the reader to get a vivid picture of participants’ obstacles and strategies. For this reason, we reinserted some statements. For example, one edited section now reads as follows (p. 14-16):  *Obstacles Experienced in Both the Anaerobic and the Aerobic Test. Effort was the most reported obstacle in both tests (e.g., “it’s getting harder and harder”). Participants stated that frustration or anger about their own performance was obstructive (e.g., “frustrated when resistance was higher than expected”). Some participants reported discomfort regarding the ergometer, were occupied with their physical reactions (e.g., “getting nauseous”), or their position during the test, which they considered hindering. Demotivation to exert oneself was perceived as an obstacle as well. Some reported to have been distracted by information on the screen or by negative thoughts. Especially in the aerobic test, not being able to distract oneself was perceived as an obstacle. Some participants stated the test was not important to them and indicated a lacking incentive for the task.* |
| **Issue 9**  Line 692 – Although this is referred to as ambition, arguably this seems to resemble achievement motivation (ego and an approach orientation) rather than ambition? | Thank you for this very interesting thought. As we did not base our qualitative analysis on certain theoretical groundworks, we would rather refrain from renaming one category with such a well-researched and important concept such as achievement motivation even though we would agree with your assessment that that some of the participants’ statements reasonably fit into this concept. Of course, we would change the category name if you and the editor think it fits significantly better. |
| **Issue 10**  Line 718 - It is not clear what is meant with anecdotal evidence? | We apologize for this inclarity. We have changed the wording and the passages now read as follows (p. 18-19):  *Comparing the goal (M = 8.3 ± 0.8) and the implementation intention condition (M = 8.9 ± 2.0) in the anaerobic test at the first session (Fig 2a) provided little evidence for differences between conditions, BF10 = 0.58. In regard to the aerobic test (Fig 2b), we found slight evidence (BF10 = 1.38) for higher time-to-exhaustion in the implementation intention (M = 24.0 ± 7.5) than in the goal intention condition (M = 19.9 ± 7.4).*  *(…)*  *This corresponds to slight and moderate evidence in favor of the Session main effect model, respectively.*  *(…)*  *In comparison, the model with the main effect of Condition received less support, BF10 = 0.62, which corresponds to little evidence in favor of the null model.* |
| **Issue 11**  Line 829-831 – I guess what would be relevant to the reader is to know how if-then plans work for less experienced athletes – i.e. what are the applied implications here? Generally a summary of applied implications for the sport psychology practitioner could be stated more clearly. | Thank you for this very important suggestion. We completely agree with you that a derivation for practical application would be helpful at this point which is why we added the following sentence (p. 21):  *In practice, it might be helpful to educate less experienced athletes about possible physiological sensations in advance and to suggest several effective then-strategies for dealing with common if-obstacles (e.g., effort, muscle pain) in order to create a helpful if-then plan (as effectively applied in other if-then planning studies in sport (45)).*  Furthermore, we summarized the applied implications in the conclusion, which now reads as follows (p. 26-27):  *In this study, we investigated the obstacles exercisers face during an anaerobic and an aerobic cycling test, as well as the strategies they considered helpful for dealing with these obstacles. We further investigated whether if-then plans based on these obstacles and strategies lead to improved performance. Qualitative analyses of interviews conducted with participants immediately after the tests revealed substantial insights into diverse sets of obstacles and strategies. The quantitative analyses suggest that an if-then planning intervention did not improve anaerobic performance or time-to-exhaustion. These findings indicate that participants were able to identify exercise-related obstacles and useful strategies; moreover, performance improvements in the aerobic test suggest that they could use this information to their advantage. However, if-then planning provided no additional benefits. When using if-then planning for less experienced athletes in practice, it might be helpful to educate exercisers about potential obstacles (e.g., muscle soreness) and to suggest potential strategies (e.g., self-encouragement), to use one if-then plan that is rehearsed with a sufficient time lag before use, and to ensure that exercisers are familiar with the sport so that they feel more self-efficient per se. Future research should complement our study with a focus on more experienced athletes and other intervention techniques. Nevertheless, our findings shed novel light onto the complex interplay of performance-related factors when investigating the impact of psychological interventions designed to help athletes in dealing with determinants of endurance performance.* |
| **Issue 12**  Line 844 - Would you say that an anaerobic test represents endurance performance? This is not typically considered as such (see also Gastin, 2001) | Thank you for pointing out this very important aspect. We clarified this in the methods section (p. 9-10):  *It is the most commonly used test when it comes to measuring anaerobic performance (53), is associated with a positive influence on endurance performance (54), and can also be used to predict endurance performance (55).*  We also changed the wording in the corresponding line to avoid misunderstandings and it now reads as follows (p. 22):  *In contrast to prior research that took a broader view on the challenges of endurance sports (e.g., comprising competitions and training; (1)), we decided to take a more narrow view by focusing on the obstacles faced during two very specific cycling performances (i.e., an anaerobic and an aerobic tests).* |
| **Issue 13**  Line 972-973 – Check sentence structure – grammar. | Thank you for your helpful correction. We have corrected the grammatical error (p. 25). |

**Our response to the comments of reviewer 2:**

| **Reviewers’ comments** | **Our response** |
| --- | --- |
| **General comments:** The authors did a good job in revising their manuscript. It is much straighter forward now and the readability has improved considerably. Furthermore, it now highlights the “concepts” trying to explain the contributing factors to the psychological determinates of endurance performance as well as the contribution of the authors results to this field. I have some minor suggestions (see below) that should be considered and afterwards the manuscript can be accepted for publication in Plos One. | Thank you for your positive evaluation of our revision and your further constructive comments below. We thankfully implemented your final feedback on our manuscript. |
| **Issue 1**  **1. Introduction:** l50-52: Please provide short examples, why this topic is of scientific and practical importance. You only state that it is, but not why. | Thank you for this important suggestion. We added examples in order to exemplify the importance of this topic (p. 3):  *The specific psychological obstacles that exercisers face during an endurance performance and the strategies they apply to deal with them requires more research as this topic is of great scientific and practical importance (1,2), for example when it comes to enhancing motivation on continuous athletic activity or exercisers’ athletic performance in general.* |
| **Issue 2**  l59, 63, …: Please add additional brackets for the references within the brackets. | Thank you for your feedback, we have added the brackets in all instances. |
| **Issue 3**  l105: Please add a comma after test. | Thank you for your correction, we have implemented the comma accordingly (p. 5). |
| **Issue 4**  l106: I suggest to use “steady” instead of “frequent”. | Thank you for this suggestion. We have corrected this instance and it now reads as follows (p. 5):  *Due to the steady increase in power output, the aerobic test probably requires significantly more self-control from the exerciser.* |
| **Issue 5**  2. Methods l170-171: Please use the same description of mean and SD. Here you have both SD in brackets and plus/minus. Please check this aspect throughout the manuscript. | We are sorry for this error. We have checked the entire manuscript to make sure everything is described consistently. |
| **Issue 6**  3. Discussion l587: I suggest to use “steady” instead of “frequent”. | Thank you again for your feedback. The sentence now reads as follows (p. 26):  *As the aerobic test requires a frequent steady increase in power output and thus poses increasing self-regulatory demands, a test that feels more achievable (e.g., a time trial) could have heightened participants’ perceptions of self-efficacy.* |
| **Issue 7**  l599: This is not only a limitation from the “exercise physiology perspective” as both physiological and psychological alterations during motor tasks are interdependent and “history-dependent”. Therefore, please adjust the wording accordingly. | Thank you for this very important input. We have changed the wording to incorporate this fact (p. 26):  *Additionally, conducting both performance tests on the same day is a limitation of this study from an exercise physiology and psychology perspective: Perceptual and physiological processes in the first test might have influenced those in the second test (e.g., in terms of residual fatigue; see (98) for a detailed review).* |
